# Acting with awareness moderates the association between lifetime exposure to interpersonal traumatic events and craving via trauma symptoms: a moderated indirect effects model

**DOI:** 10.1186/s12888-022-03931-1

**Published:** 2022-04-22

**Authors:** Gladys E. Ibañez, Mariana Sanchez, Karina Villalba, Hortensia Amaro

**Affiliations:** 1 Department of Epidemiology, Robert Stempel College of Public Health and Social Work, Florida International University, Florida Miami, US; 2 Department of Health Promotion and Disease Prevention, Robert Stempel College of Public Health and Social Work, Florida International University, Florida Miami, US; 3 Department of Population Health, College of Medicine, University of Central Florida, Florida Miami, US; 4 Herbert Wertheim College of Medicine and Robert Stempel College of Public Health, Florida International University, Florida Miami, US

**Keywords:** Trauma exposure, Substance use, Craving, Mindfulness, Women, Developmental timing, Acting with awareness

## Abstract

**Background:**

History of exposure to traumatic events (ETE) is common among women in substance use disorder (SUD) treatment and is related to craving. We examined whether ETE (i.e., emotional, physical, sexual abuse) in childhood, adulthood, or both is related to craving via trauma symptoms and how trait mindfulness might attenuate this association.

**Methods:**

Baseline data from a larger randomized clinical trial of a mindfulness-based intervention for women (*N* = 245) in SUD treatment were used. Inclusion criteria were: 18–65 years of age, SUD diagnosis, English fluency, no cognitive impairment, and willingness to be audio recorded and provide consent. Demographics and validated measures of ETE, posttraumatic stress symptoms, trait mindfulness, and substance use craving were collected via in-person interviews. Descriptive statistics, correlational analysis, and relative direct, indirect, and conditional indirect effects models were run.

**Results:**

Most participants identified as Hispanic (58.5%), had at least a high school education (52.2%), with a mean age of 32.2. Women reported ETE in childhood only (20.4%), adulthood only (17.5%), both childhood and adulthood (50.0%), and never (11.4%). Compared to women with ETE in both childhood and adulthood, those with exposure in adulthood only (β = -.10, 95% CI = -.20, -.02) or no exposure (β = -.11, 95% CI = -.23, -.03; [*∆R*_2=_ *.347, F*(8, 245) = 15.7, *p* < .001) had lower craving via lower trauma symptomatology but no difference when compared to those with ETE only in childhood. Acting with awareness moderated this indirect effect (*∆R*_2_ = .04, *F*(3, 245) = 4.66, *p* = .004. At low levels of awareness, women with ETE during both childhood and adulthood reported higher craving via trauma symptomatology than women with no exposure or only adulthood exposure.

**Conclusions:**

Low levels of acting with awareness may worsen trauma symptoms after ETE, which in turn may lead to more craving for women in substance use treatment. Despite a small moderating effect size, acting with awareness may have clinical significance due to the prevalence of trauma symptoms among women in SUD treatment.

## Background

History of exposure to traumatic events (ETE) such as abuse and interpersonal violence in childhood and adulthood are common among people with substance use disorders (SUD) [1–3]. Studies of individuals in SUD treatment have indicated important gender differences in types of trauma exposure, with women reporting experiences of sexual and physical abuse [4] and childhood traumatic events [5] more often than men. In turn, ETE has been associated with poorer SUD treatment outcomes [6–9].

Implicated in this relationship is the role of craving, which is a major predictor of treatment dropout [10–12] and relapse [13–17]. Women with SUD report significantly higher craving than their male counterparts [18] and sensitivity to craving in the presence of stress [19] and drug cues [20]. Thus, history of ETE is important for women in SUD treatment, because such exposures are associated with greater degrees of craving [21, 22].

### Theoretical rationale

Witkiewitz & colleagues [23, 24] proposed a model of relapse, which includes craving as a key factor, and one that focused on *tonic* and *phasic* processes. Tonic processes are underlying (relatively fixed) factors that make a person vulnerable to problematic behaviors [23] such as ETE. Phasic processes refer to more immediate risk factors within a certain situation that may increase a person’s vulnerability to use again [23] such as current trauma symptomatology and craving. Trait mindfulness, an inherent trait that people possess to varying degrees regardless of mindfulness practice, is related to psychological health [25], and which can be improved with mindfulness training [26]. In Witkiewitz, et al.’s model, mindfulness can potentially moderate the association between tonic and phasic processes. In addition, although the literature on developmental timing of the trauma exposure is very limited, a few recent studies show that the timing of trauma exposure is related to risk factors (e.g., emotional dysregulation [27]) that may create vulnerability to substance use. Childhood trauma, for example, is known to lead to issues with information processing that involve over identification to threats, and emotional processing linked to emotional dysregulation and elevated emotional reactivity [28]. These changes in social information and emotional processing lead to future psychopathology [28], and creates further vulnerability for those who experience subsequent adulthood trauma. Therefore, those who experience childhood trauma may lack the coping resources needed to deal with adulthood traumas. The present study adapts Witkiewitz and colleagues’ model to examine the mechanism between developmental timing of trauma exposure (i.e., a tonic process) and trauma symptomatology and craving (i.e., phasic processes); and, because trait mindfulness is an inherent trait already present during trauma exposure, whether trait mindfulness modifies the pathway between the developmental timing of trauma exposure and craving via trauma symptomatology.

### Trauma exposure

ETE is defined as experiencing a serious injury or witnessing a serious injury to or the death of someone else, facing imminent threats of serious injury or death to self or others, or experiencing a violation of personal physical integrity [29]. ETE can be brief and specific to a time and place or chronic—experienced over long periods [29]. In this paper, we consider three types of ETE (i.e., physical, sexual, and emotional abuse), also referred to as interpersonal traumas, and their occurrence at different developmental stages (i.e., childhood only, adulthood only, or both). We focus on exposures to interpersonal trauma because it is common among women seeking substance use treatment [8], and its association with substance use disorder [30], and with severity of substance use [31]. To the authors’ knowledge, there are no studies that investigate the association between interpersonal trauma and craving. Moreover, while substance use disorder is related to a posttraumatic stress disorders (PTSD) diagnosis [32], trauma symptoms among those exposed to traumatic events but who do not meet criteria for PTSD diagnosis are also associated with substance use [33]. It is important to study trauma symptomatology in terms of prevention and overall well-being for those in SUD treatment who may or may not meet a PTSD diagnosis.

Women in substance use treatment are more likely to have experienced sexual abuse [8], physical abuse [4], and childhood traumatic events compared to their male counterparts [5]. Most of the literature on the consequences of ETE has focused on specific lifespan periods, primarily childhood [22, 31, 34, 35], or cumulatively during the lifetime without distinctions regarding when the exposure occurred [36–39]. Few studies have examined trauma experienced in adulthood only [40]. Developmental timing of ETE is an important predictor of substance use [41] and correlate of substance use [27]. Yet to the authors’ knowledge, no studies have examined developmental timing of ETE and its relationship to craving. This lack of distinction or comparison of ETE across lifespan periods has resulted in an incomplete understanding of the relationship between timing of ETE and substance use- and craving-related sequalae. For example, studies on the substance use effects of ETE in adulthood have generally not considered previous exposures in childhood [42], and studies of adult women’s history of childhood exposures and the relationship of these to substance use-related factors have not considered ongoing exposures in adulthood [43]. Both represent gaps in knowledge regarding the distinctive and potentially synergistic impacts of ETE at various developmental periods on substance use-related outcomes such as craving.

### Craving

Craving, or the urge to use substances, is defined as an impulsive drive, emotional state, or response to a stressor [44]. Craving is a common clinical feature among individuals with SUD and a significant predictor of substance use and relapse following treatment for SUD [17]. When an individual with a history of SUD is confronted with a stressor in the absence of adequate coping skills, this encounter may elicit craving, leading to substance use [45, 46]. In addition, recent studies found that trauma exposure during childhood predicted craving in women with opioid use disorder [21]. One explanation is self-medication theory, which proposes that among people with a history of trauma exposure, alcohol or drug use serves as a coping response to manage symptoms of stress and anxiety, eventually leading to the onset of SUDs [47]. Moreover, according to the neurobiology literature, stress-related factors such as early adverse life events, trauma exposure, and prolonged and chronic stress experiences significantly alter the neurobiological pathways responsible for stress regulation and cognitive and behavioral control, which leads to craving and subsequent drug use [44, 48, 49].

### Trait mindfulness

Dispositional mindfulness, or trait mindfulness, is a multidimensional construct [50, 51] that refers to an individual’s tendency to maintain awareness in a nonjudgmental and nonreactive way to present situations [52]. Trait mindfulness is thought to be composed of five facets: ability to observe internal and external experiences (observe), acting with awareness in the present moment (awareness), ability to describe internal experiences (describe), not judging inner experiences (nonjudge), and letting go of thoughts and feelings rather than reacting (nonreactivity) [52, 53]*.* Previous studies found that among women with SUD, trait mindfulness is associated with lower trauma symptomatology [54, 55] and greater psychological adjustment following a trauma exposure [56].

### Trait mindfulness as a moderator

Trait mindfulness is characterized by nonreactive awareness and acceptance which may serve as a protective factor and mitigate the co-occurrence of trauma and addiction [50, 57]. For example, in associations between trauma exposure and anxiety [58], perceived stress and psychological symptoms [59], future time perspective and depression [60], and adverse childhood events and adult health [61]. A study has also shown that trait mindfulness was significantly inversely associated with post-traumatic stress symptoms [62]. It is well established that exposure to traumatic events may trigger post-traumatic stress disorder (PTSD), which is characterized by recurrent, distressing symptoms associated with the original trauma [63, 64]. Thus, instead of avoiding or denying such experiences, trait mindfulness teaches individuals to cope with stressful life events by adopting an attitude of self-compassion and nonjudgment towards their own thoughts and emotions [65]. Other studies found that certain facets of mindfulness such as acting with awareness and nonreactivity, moderate health outcomes [66, 67]. To the authors’ knowledge, no published study has examined trait mindfulness as a moderator of the association between ETE, trauma symptomatology, and craving.

### The present study

The present study examines the distinction between ETE in childhood, adulthood, or both; assesses its direct and indirect effects on craving; and examines trait mindfulness, and its five facets, as a moderator among women in a residential SUD treatment program. Given the scant attention on mindfulness as a moderator of ETE and craving via trauma symptomatology, and the increasing use of mindfulness training interventions in SUD treatment, this study attempts to better understand the role of mindfulness in ETE and related symptomatology and how it relates to craving among women in treatment for SUD. The proposed study conducted a moderated indirect-effects analysis to examine the following hypotheses: (1) Women who report both childhood and adulthood ETE will report more trauma symptoms relative to those who report childhood only, adulthood only, or no trauma exposure. (2) Trait mindfulness will moderate the association between timing of ETE and trauma symptoms, wherein this association will be weaker for those with high level of mindfulness. Specifically, the association between ETE in both childhood and adulthood and trauma symptoms will be weaker for those with high mindfulness relative to those whose trauma exposures occur in childhood only or adulthood only or who have no trauma exposure. (3) Trauma symptoms will be positively related to craving. (4) ETE that occurs in both childhood and adulthood will have significant direct and indirect effects on craving relative to exposure in childhood only or adulthood only or no trauma exposure. (5) Trait mindfulness will moderate the indirect effects of ETE on craving via trauma symptoms. Specifically, at low levels of trait mindfulness, the association between ETE and craving via trauma symptoms for women who experienced trauma in both childhood and adulthood will be stronger than for women who experienced trauma in childhood only or adulthood only or no trauma. At high levels of trait mindfulness, the association between ETE via trauma symptoms on craving will not differ between trauma exposure groups.

## Method

Data for the present study came from a larger randomized clinical trial of Moment-by-Moment in Women’s Recovery**,** a mindfulness-based relapse prevention adjunctive intervention program for women (*N* = 245) in SUD treatment [68, 69]. Data in the present analyses came from study site clinical patient records and selected validated measures administered during a baseline in-person structured interview prior to randomization and intervention. Participants in the parent study were recruited at entry into a gender-specific residential SUD treatment program. Inclusion criteria for the research trial were as follows: client at the residential treatment study site, female, adult aged 18 to 65 years, diagnosed with SUD via clinical record, fluent in English, and agreed to participate in the study. Exclusion criteria were as follows: inability to comprehend or sign informed consent, cognitive impairment, untreated psychotic disorder or severe chronic mental health condition based on clinical intake LR-DSM-IV or DSM-V diagnostic assessment, older than 65 years because this was unusual at the site, reported suicidality during the prior 30 days based on clinical intake assessment, current prisoner, more than 6 months pregnant, and not willing to sign a HIPAA form or be audio recorded during interviews and intervention sessions. All parent study procedures were approved by the affiliated university’s institutional review board. Intervention results of the parent study are available elsewhere [69, 70]. Data were collected via Research Electronic Data Capture computer-assisted interviews by trained researchers during in-person meetings. All baseline interviews were conducted at the study site.

### Measures

#### Exposure to traumatic events

The Life Stressor Checklist-Revised [71] is a 30-item measure of the occurrence of traumatic events and stressors (with response options of yes or no) that has been validated in women with co-occurring SUD and mental health disorders and histories of interpersonal violence victimization [72]. We used items assessing exposure to sexual, physical, and emotional traumatic experiences and their timing. Four categories of ETE were created: no exposure = 0 (no trauma exposure); only up to age 17 but none thereafter = 1 (childhood only); only after age 17 = 2 (adulthood only); and exposure both before and after age 17 = 3 (both childhood and adulthood, which was the referent group).

#### Trauma symptomatology

Trauma symptom severity in the last 30 days was assessed with the Posttraumatic Symptom Scale–Self-Report [73]. The scale measures severity of trauma symptoms on a 4-point Likert scale, ranging from *not at all* (0) to *almost always* (3). A total scale score was used to represent total trauma symptom severity. The scale demonstrated good internal consistency in the study sample (α = 0.94).

#### Alcohol and drug craving

The Penn Alcohol Craving Scale [74], a 5-item measure, was adapted to include craving for both alcohol and other drugs. The scale measures frequency, intensity, and duration of craving during the past 30 days. The response format is a 6-point Likert scale (0 = *never* to 6 = *nearly all of the time*). Average scale scores were used, and internal consistency in the current sample was excellent (α = 0.93).

#### Trait mindfulness

Trait mindfulness was measured using the short 24-item version of the Five-Facets Mindfulness Questionnaire [53], which assesses an individual’s tendency to be mindful in everyday life. Items are rated on a 5-point Likert scale, ranging from *never true* (1) to *very often true* (5). In addition to a total sum score, it provides sum scores for the following mindfulness dimensions: observing (four items), describing (five items), acting with awareness (five items), nonjudgmental acceptance (five items), and nonreactivity (five items). Internal consistency (Cronbach’s alpha) for each subscale in this sample were as follows: observing = 0.73, describing = 0.73, acting with awareness = 0.81, nonjudgmental acceptance = 0.74, and nonreactivity = 0.66. The total score as well as the sum score for each of the facets will be examined as possible moderators.

## Covariates

### Alcohol and drug severity

An adapted Addiction Severity Index-Lite [75] was used to evaluate alcohol and drug use severity in the 30 days prior to entering treatment, scored in accordance with the published manual [76]. Specifically, participants were asked about money spent on alcohol or drugs and the number of days they experienced alcohol or drug problems in the 30 days prior to treatment entry. Further, they reported their subjective evaluation of how bothered they were by these drug and alcohol problems and how important they considered treatment to addressing these problems on a 5-point Likert scale ranging from 0 (*not at all*) to 4 (*extremely*).

### Demographics

The following demographic variables were assessed as potential covariates: age, race and ethnicity (1 = *non-Hispanic White*, 2 = *non-Hispanic Black*, 3 = *Hispanic*), education (1 = *less than high school*, 2 = *high school or equivalent*, 3 = *some postsecondary education*), and housing status in the past 8 months (1 = *homeless*, 2 = *unstable accommodation*, 3 = *institution*, 4 = *own place*, 5 = *someone else’s place*).

### Data analysis plan

Descriptive statistics (means, standard deviations, skewness, kurtosis) were calculated to describe sample characteristics and test for normality assumptions for all continuous variables. Bivariate analysis of demographic and key variables was conducted utilizing one-way analyses of variance or chi-square tests. Zero-order correlations were conducted for all continuous variables. Demographic and other continuous variables significantly related at the bivariate level to trauma symptomatology and craving were included in subsequent models as covariates.

All models were run using PROCESS v3.2 for SPSS 25. Primary data analysis included examining relative indirect effects, relative moderation effects, and relative conditional indirect effects. In the relative indirect-effects models, we examined the relative effects of developmental timing of ETE (i.e., no trauma, childhood trauma only, adulthood trauma only, and both childhood and adulthood trauma) both directly and indirectly on craving via trauma symptomatology. The referent group was participants with both childhood and adulthood trauma. Confidence intervals that do not include zero were considered significant [77]. Post hoc analyses were conducted to examine all possible pairwise comparisons across trauma exposure groups by switching the referent groups accordingly. A Bonferroni approach to multiple test correction was utilized to adjust for the multiple comparisons with a criterion *p*-value of 0.012.

In the moderation models, we examined whether trait mindfulness and its five facets moderated the relative association between ETE and trauma symptoms. In addition to analyzing the total trait mindfulness scale as a moderator, separate models were run using each distinct trait mindfulness subscale (observing, describe, awareness, nonjudgment, nonreactivity). Finally, a relative conditional indirect-effects model was conducted to examine whether trait mindfulness moderated the indirect-effects model. That is, did the level of trait mindfulness (low [i.e., 1SD below the mean], mean, or high [i.e., 1SD above the mean]) moderate the relative indirect effect between the distinct trauma exposure groups and craving via trauma symptoms? For the moderated indirect-effects model on craving, we examined only trait mindfulness factors (overall or subscales) that were significant moderators in the previous moderation model. An *index of moderated indirect effects* was used to examine whether the conditional indirect effects significantly differed across levels of trait mindfulness. The index of moderated indirect effects was an inferential test to evaluate whether the extent of moderated indirect effects was statistically different than zero [78]. No missing data were present for the variables used in the primary analyses.

## Results

### Descriptive findings

As shown in Table 1, participants had a mean age of 32.2 and most identified as Hispanic (58.5%), had never married (74.3%), had at least a high school education (52.2%), and in the 8 months prior to treatment entry had used methamphetamines (77.6%), marijuana (55.9%), alcohol until intoxication (50.6%), and 53.5% reporting daily polydrug use. All were diagnosed with SUD; 76.2% were diagnosed with drug use disorder only, 10.0% with alcohol use disorder only, and 13.8% with alcohol and drug used disorder. Overall, most women reported at least one interpersonal ETE (87.8%). Regarding trauma exposure groups, 50.2% reported both childhood and adulthood exposure, 20.8% reported childhood exposure only, 16.7% reported adulthood exposure only, and 12.2% reported no exposure. The most common exposure type in childhood was emotional (54.3%), followed by sexual (46.1%) and physical (36.3%), and the most common exposure type in adulthood was physical (47.3%), followed by sexual (39.6%) and emotional (13.1%).

**Table 1:** Sample Characteristics (*N* = 245)

Variables	Exposure to Traumatic Events				
	Total	No Exposure	Childhood Only	Adulthood Only	Childhood and Adulthood
	(*N* = 245)	(*n* = 30)	(*n* = 51)	(*n* = 41)	(*n* = 123)
Age, *M* (*SD*)	32.2 (8.9)	30.8 (8.3)	29.6 (6.0)	34.2 (9.8)	33.0 (8.9)
Race, *n* (%)^a^					
Hispanic	141 (58.5)	18 (60.0)	34 (68.0)	22 (55.0)	67 (55.4)
Non-Hispanic White	50 (20.7)	4 (13.3)	10 (20.0)	7 (17.5)	29 (24.0)
Non-Hispanic Black	50 (20.7)	8 (26.7)	6 (12.0)	11 (27.5)	25 (20.7)
Education, *n* (%)					
< High school	117 (47.8)	16 (53.3)	27 (52.9)	20 (48.8)	54 (43.9)
Completed high school	67 (27.3)	7 (23.3)	11 (21.6)	8 (19.5)	41 (33.3)
Some education after high school	61 (24.9)	7 (23.3)	13 (25.5)	13 (31.7)	28 (22.8)
Housing status in 8 months prior to treatment, *n* (%)					
Homeless	56 (22.9)	7 (23.3)	10 (19.6)	10 (24.4)	29 (23.6)
Unstable accommodations	22 (9.0)	1 (3.3)	2 (3.9)	6 (14.6)	13 (10.6)
Institution	43 (17.6)	8 (26.7)	10 (19.6)	9 (22.0)	16 (13.0)
Own place	44 (18.0)	3 (10.0)	9 (17.6)	5 (12.2)	27 (22.0)
Someone else’s place	80 (32.7)	11 (36.7)	20 (39.2)	11 (26.8)	38 (30.9)
Child custody, *n* (%)					
No minor children in custody	117 (47.8)	17 (56.7)	32 (62.7)	15 (36.6)	53 (43.1)
Minor children in custody	84 (34.3)	9 (30.0)	15 (29.4)	16 (39.0)	44 (35.8)
No children under 18	19 (7.8)	2 (6.7)	1 (2.0)	4 (9.8)	12 (9.8)
No children	25 (10.2)	2 (6.7)	3 (5.9)	6 (14.6)	14 (11.4)
Marital status, *n* (%)					
Never married	182 (74.3)	24 (80.0)	42 (82.4)	29 (70.7)	87 (70.7)
Separated, divorced, or widowed	46 (18.8)	4 (13.3)	5 (9.8)	10 (24.4)	27 (22.0)
Married	17 (6.9)	2 (6.7)	4 (7.8)	2 (4.9)	9 (7.3)
Alcohol addiction severity, *M* (*SD*)	.14 (.21)	.08 (.18)	.15 (.24)	.11 (.18)	.15 (.21)
Drug addiction severity, *M* (*SD*)	.15 (.14)	.10 (.12)	.17 (.15)	.12 (.10)	.17 (.14)
Top substance used in 8 months prior to treatment entry, *n* (%)^b^					
Methamphetamines	190 (77.6)	25 (83.3)	42 (82.4)	27 (65.9)	96 (78.0)
Marijuana	137 (55.9)	18 (46.2)	26 (60.5)	15 (46.9)	78 (59.5)
Alcohol to intoxication	124 (50.6)	10 (33.3)	30 (58.8)	18 (43.9)	66 (53.7)
Cocaine or crack Polydrug (> 1 drug used per day)	33 (13.5) 131 (53.5)	4 (13.3) 12 (42.9)	2 (3.9) 26 (52.0)	7 (17.1) 20 (46.5)	20 (16.3) 73 (58.9)
Substance use disorder, *n* (%)^c^					
Drug use disorder only	182 (76.2)	21 (72.4)	39 (79.6)	25 (64.1)	97 (79.5)
Alcohol use disorder only	24 (10.0)	2 (6.9)	6 (12.2)	3 (7.7)	13 (10.7)
Both	33 (13.8)	6 (20.7)	4 (8.2)	11 (28.2)	12 (9.8)
Mental health diagnosis other than SUD, *n* (%)^c^	138 (57.7)	15 (51.7)	32 (65.3)	20 (51.3)	71 (58.2)
Post-traumatic stress disorder (PTSD) diagnosis, *n* (%)	65 (26.5)	6 (21.4)	15 (30.0)	14 (32.6)	30 (24.2)
Depressive disorder diagnosis, *n* (%)	40 (16.3)	4 (14.3)	4 (8.0)	6 (14.0)	26 (21.0)
Other mental health diagnosis, *n* (%)	66 (26.9)	11 (39.3)	11 (22.0)	11 (25.6)	33 (26.6)

Percentages are valid percentages

^a^Data not included for “other” category (*n* = 4)

^b^Percentages add up to more than 100% due to use of multiple substances

^c^Missing data (*n* = 6)

### Trauma exposure groups

Preliminary data analysis showed that all key variables were normally distributed. No significant differences across trauma exposure groups were found in age, alcohol use severity, drug use severity, race and ethnicity, education, or housing (Table 1).

### Craving

There were racial and ethnic differences in craving: *F*(2, 238) = 6.23, *p* < 0.002. Both non-Hispanic White women (*M* = 2.52, *SD* = 1.89) and Hispanic women (*M* = 2.63, *SD* = 1.70) reported more craving than non-Hispanic Black women (*M* = 1.66, *SD* = 1.41). Alcohol addiction severity (*r* = 0.34, *p* < 0.01) and drug addiction severity (*r* = 0.52, *p* < 0.01) were both positively correlated with craving (see Table 2). No significant differences in craving by age, education, housing, or trauma exposure groups were found.

**Table 2:** Correlations for Key Observed Variables (*N* = 245)

Variable	*M*	*SD*	1	2	3	4	5	6	7	8	9	10
1. Craving	2.42	1.72										
2. Age	32.21	8.87	-.05									
3. Alcohol severity	.14	.21	.34**	.12								
4. Drug severity	.15	.14	.52**	-.09	.24**							
5. PTSD	17.69	12.71	.34**	-.04	.28**	.30**						
Mindfulness												
6. Total	76.69	13.15	-.29**	.01	-.13*	-.31**	-.35**					
7. Describe	16.51	4.31	-.22**	.04	-.13*	-.28**	-.28**	.79**				
8. Nonreactivity	14.93	3.94	-.18**	.05	-.10	-.09	-.20**	.53**	.35**			
9. Observe	13.75	3.95	-.15*	.11	-.02	-.10	.01	.56**	.39**	.52**		
10. Nonjudgment	14.67	4.42	-.10	-.07	-.03	-.21**	-.19**	.46**	.22**	-.18**	-.20**	
11. Awareness	16.83	4.73	-.23**	-.08	-.11	-.25**	-.39**	.72**	.47**	.05	.13*	.46**

*M* mean, *SD* standard deviations, *PTSD* posttraumatic stress disorder symptomatology

^*^p < .05. **p < .01 (two-tailed)

### Trauma symptomatology

There were significant differences in trauma symptomatology across trauma exposure groups: *F*(3, 241) = 10.12, *p* < 0.001. Women who reported both childhood and adulthood traumas reported higher trauma symptomatology (*M* = 21.01, *SD* = 12.78) than women who reported no trauma (*M* = 10.13, *SD* = 9.32) and adulthood trauma only (*M* = 12.07, *SD* = 11.83). Women who reported exposure to traumatic events in childhood only (*M* = 18.63, *SD* = 11.78) reported more trauma symptomatology than women with no exposure to traumatic events and those with exposure in adulthood only. Severity of alcohol addiction (*r* = 0.28, *p* < 0.001) and severity of drug addiction (*r* = 0.30, *p* < 0.001) were positively related to trauma symptomatology (see Table 2). No significant differences in trauma symptomatology by age, race and ethnicity, education, or housing were found. Zero-order bivariate correlations of all key continuous variables are shown in Table 2.

### Trait mindfulness

There were differences in the describing facet of mindfulness by education: *F*(2, 242) = 5.48, *p* < 0.005. Participants with more than a high school education (*M* = 18.02, *SD* = 4.37) reported higher levels of describing than women with less than a high school education (*M* = 15.81, *SD* = 4.47). There were also racial and ethnic differences in total trait mindfulness (*F*[2, 238] = 5.26, *p* < 0.006), describing (*F*[2, 238] = 4.65, *p* < 0.010), and observing (*F*[2, 238] = 6.26, *p* < 0.002). Hispanic women reported lower levels of total trait mindfulness (*M* = 74.42, *SD* = 12.60) than non-Hispanic White (*M* = 79.50, *SD* = 14.79) and non-Hispanic Black (*M* = 80.20, *SD* = 11.37) women; lower levels of observing (*M* = 12.99, *SD* = 4.15) than non-Hispanic White (*M* = 14.96, *SD* = 4.16) and non-Hispanic Black (*M* = 14.56, *SD* = 3.57) women; and lower levels of describing (*M* = 15.84, *SD* = 4.03) than non-Hispanic White women (*M* = 17.66, *SD* = 4.96).

Based on these bivariate analyses, we included age, race and ethnicity, alcohol severity, and drug severity as covariates in all subsequent models.

### Indirect effects analysis

Hypotheses 1, 3, and 4 posited that women who report both childhood and adulthood trauma exposure will report more trauma symptoms relative to those who report child only, adult only, or no trauma exposure; trauma symptoms will be positively related to craving; and exposure to trauma that occurs in both childhood and adulthood will show significant direct and indirect effects via trauma symptomatology on craving relative to other trauma exposure groups.

**Fig. 1:**
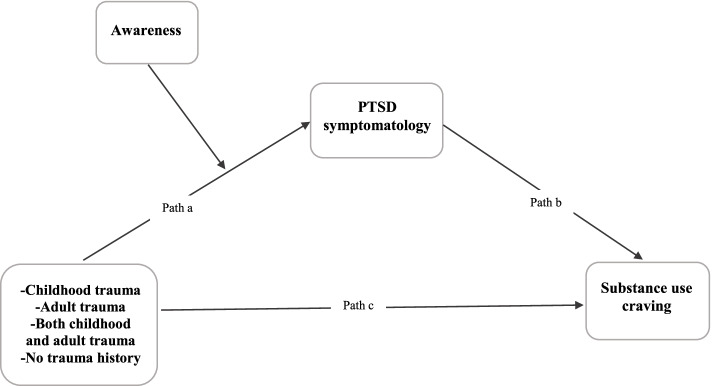
Heuristic model of the relative conditional indirect effects on craving

The first study aim was to examine the relative direct and indirect effects of trauma exposure on craving via trauma symptomatology. Results indicate significant relative direct effects of trauma exposure on trauma symptomatology (see path a in Fig. 1). Specifically, lower levels of trauma symptomatology were found among women with no history of ETE (β = -0.69, *p* < 0.001) and only adult history of ETE (β = -0.58, *p* < 0.001) compared to those with ETE during both childhood and adulthood. No other group differences in trauma symptomatology were found. Trauma symptomatology was directly associated with higher levels of craving (β = 0.16, *p* = 0.01; see path b in Fig. 1). No relative direct effects across trauma exposure groups were found on craving (see path c in Fig. 1). Indirect-effects analysis indicated significant relative indirect effects of trauma symptomatology on craving by trauma exposure group. That is, compared to women exposed in both childhood and adulthood, those with no exposure (β = -0.11, 95% CI = -0.23, -0.03) and exposure in adulthood only (β = -0.10, 95% CI = -0.20, -0.02) had lower levels of substance use craving resulting from lower trauma symptomatology. Compared to women exposed to traumatic events during childhood, those with no trauma history (β = -0.08, 95% CI = -0.19, -0.01) and only adulthood trauma (β = -0.06, 95% CI = -0.16, -0.00) had lower levels of substance use craving resulting from lower trauma symptomatology. Lastly, results of the mediation analyses indicate that 20.9% of the variance in trauma symptomatology was explained by exposure to traumatic events, while all predictor variables in the full model accounted for 34.7% of the variance in craving [*∆R*_2=_ *0.347, F*(8, 245) = 15.7, *p* < 0.001]. No relative indirect effects were found between women with ETE in both childhood and adulthood and those with ETE in childhood only.

### Moderation analysis

Hypothesis 2 posited that trait mindfulness will moderate the association between timing of exposure and trauma symptoms, wherein this association will be stronger for those with low level of mindfulness. Specifically, the association between ETE in both childhood and adulthood and trauma symptoms will be stronger for those with low mindfulness relative to those whose ETE occurs in childhood only, adulthood only, or who have no trauma exposure.

The second study aim was to examine the moderating effects of trait mindfulness on the association between trauma exposure and trauma symptomatology and craving. Models were conducted using total mindfulness and each facet of mindfulness separately. To calculate awareness levels, simple slopes were calculated using the “pick-a-point” approach with 16th, 50th, and 84th percentiles representing low, average, and high levels of awareness, respectively. Trait mindfulness total scale score was not a significant moderator between trauma exposure and trauma symptomatology; however, significant interaction effects were found for the mindfulness facet of acting with awareness. Specifically, relative significant interactions effects were found between women with no trauma exposure (*b* = -34.81, *p* < 0.001), childhood only trauma exposure (*b* = -17.32, *p* = 0.01), and adulthood only trauma exposure (*b* = -20.32, *p* = 0.004), compared to those who experienced ETE in both childhood and adulthood, [*∆R*_2_ = 0.04, *F*(3, 245) = 4.66, *p* = 0.004]. No other relative interaction effects were found between groups. Results indicate that at low levels of awareness, women with ETE in both childhood and adulthood reported greater trauma symptomatology compared to all other trauma exposure categories. As awareness levels increased, this association decreased, such that no difference in trauma symptomatology was found across trauma exposure groups among women with high levels of awareness. Conditional effects of the trauma exposure categories on trauma symptomatology at low, average, and high levels of awareness are presented in Table 3.

**Table 3:** Relative Conditional Effects of Trauma Exposure on Trauma Symptomatology by Level of Awareness (*N* = 245)

Trauma Exposure Group^a^	*b*	*SE*	LLCI	ULCI
Low awareness				
No trauma	-18.06	3.77	**-25.47**	**-10.62**
Childhood only	-7.65	2.77	**-13.11**	**-2.20**
Adulthood only	-11.30	3.21	**-17.62**	**-4.98**
Average awareness				
No trauma	-7.39	2.20	**-11.74**	**-3.05**
Childhood only	-1.50	1.88	-5.22	2.21
Adulthood only	-5.56	1.95	**-9.40**	**-1.72**
High awareness				
No trauma	-1.30	3.00	-7.21	4.60
Childhood only	2.02	2.83	-3.55	7.83
Adulthood only	-2.28	2.61	-7.43	2.86

Coefficients are presented in unstandardized form; *LLCC* lower-level confidence interval, *ULCI* upper-level confidence intervals. Bold indicates significance at *p* < .05 TK

^a^Reference group was both childhood and adulthood trauma

**Table 4:** Results indicating relative conditional indirect effects trauma exposure on craving via PTSD symptomatology by levels of awareness (*n* = 245)

Trauma exposure category^a^	*b*	Boot *SE*	LLCI	ULCI
No trauma → PTSD → craving				
Low awareness	-.41	.18	**-.80**	**-.11**
Average awareness	-.17	.08	**-.35**	**-.04**
High awareness	-.03	.07	-.18	.11
Index of moderated mediation	.03	.02	**.01**	**.07**
Child trauma only → PTSD → craving				
Low awareness	-.17	.10	**-.40**	**-.02**
Average awareness	-.03	.05	-.15	.06
High awareness	.05	.08	-.09	.22
Index of moderated mediation	.02	.01	-.0005	.05
Adult trauma only → PTSD → craving				
Low awareness	-.25	.14	**-.56**	**-.05**
Average awareness	-.12	.07	**-.29**	**-.02**
High awareness	-.05	.06	-.20	.07
Index of moderated mediation	.02	.01	**.0002**	**.04**

Coefficients are presented in unstandardized form. Boot SE Bootstrapped standard error, LLCC lower-level confidence interval, ULCI upper-level confidence intervals. Bold indicates statistical significance at *p* < .05

^a^Reference group was both childhood and adulthood trauma → PTSD → craving

### Relative conditional indirect-effects analysis

Hypothesis 5 posited that trait mindfulness will moderate the indirect effects of trauma exposure on craving via traumatic symptoms. Specifically, at low levels of trait mindfulness, the association between trauma exposure and craving via trauma symptoms for women with ETE in both childhood and adulthood will be stronger than for women with ETE in childhood only, ETE in adulthood only, or no trauma exposure. At high levels of trait mindfulness, association between trauma exposure and craving via trauma symptoms will not differ between trauma exposure groups.

**Fig. 2:**
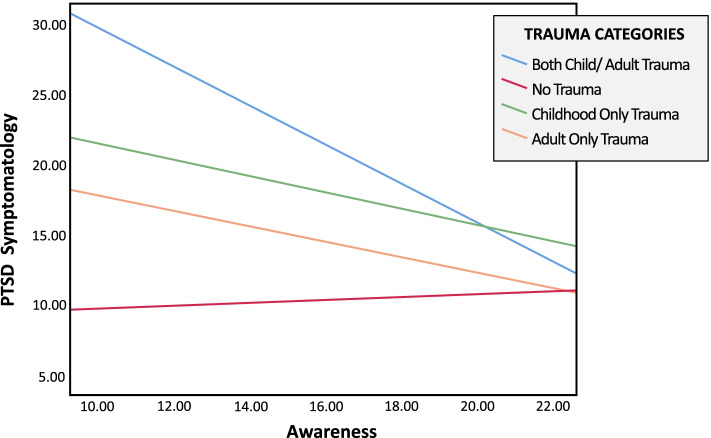
Relative conditional moderating effects

The *index of moderated indirect effects* was used to examine whether the conditional indirect effects of trauma exposure on craving via trauma symptoms differed significantly across levels of acting with awareness. Table 4 presents the results of the conditional indirect effects model. Specifically, compared to women with ETE in both childhood and adulthood, those with no trauma exposure and those with ETE in adulthood only had lower levels of substance use craving resulting from lower trauma symptomatology. These indirect effects were found among women with low and average levels of awareness. No differences in relative indirect effects were found between trauma exposure groups when levels of awareness were high. No significant moderated relative indirect effects were found between women with ETE in both childhood and adulthood compared to those with ETE in childhood only (see Fig. 2).

## Discussion

### *ETE in both childhood and adulthood has relative indirect effects on craving *via* trauma symptoms*

This is the first study to examine the developmental timing of ETE (i.e., emotional, physical, and sexual abuse), its association to craving via trauma symptomatology, and how this association may be moderated by mindfulness among women in substance use treatment. Craving is a strong predictor of relapse following residential SUD treatment [79] and therefore, it represents an important intervention target. A better understanding of factors that inform interventions to reduce and manage craving is needed. Our findings show a medium effect size for the mediation model in which developmental timing of ETE was indirectly related to craving via its association with trauma symptoms. Women who reported ETE in both childhood and adulthood relative to the other trauma exposure groups reported more trauma symptoms, which in turn was associated with a greater level of craving. This implies that women with ETE during both childhood and adulthood may be at particular risk of experiencing craving.

We know from the neurobiology literature that ETE causes neurobiological changes in brain functioning, including areas of the brain responsible for self-control [49] and behavioral and cognitive responses to stress [80, 81]. In other words, due to earlier trauma, the brain systems responsible for controlling intense emotions are deactivated and therefore, when stressors are reexperienced, the emotional reaction is elevated and accompanied by a lack of emotional and behavioral control [80, 82]. Exposure to stressors especially ones that cannot be controlled, such as childhood traumas, can lead to an overactivation of the stress response and a blunted stress response to any later trauma in adulthood [83–85]. This could explain why women who have ETE in both childhood and adulthood report more trauma symptoms and a greater level of craving than women with other ETE histories.

Our findings suggest that the developmental timing of ETE is important to consider, and that experiencing ETE in both childhood and adulthood is associated with poorer outcomes compared to those who experience no ETE or exposure only in adulthood. Unfortunately, the literature on the developmental timing of trauma is limited. However, one study found that the developmental timing of ETE was related to emotional dysregulation, an important correlate of craving, even when controlling for the frequency of exposure [27]. We cannot assess whether there was a synergistic effect produced from cumulative trauma in both childhood and adulthood. Therefore, it cannot be determined whether the association between ETE and craving is primarily due to the frequency of ETE, as cited by much of the cumulative trauma literature, or the developmental timing of ETE. Future research should investigate the neurological and cognitive impacts of childhood ETE and the related pathways through which they affect the ability to cope with subsequent ETE in adulthood. A more nuanced assessment of trauma exposure is needed to understand the different ways that trauma exposure can influence craving and other substance use outcomes.

### Acting with awareness moderates the association between ETE and trauma symptoms

The acting with awareness facet of mindfulness was a significant moderator between ETE and trauma symptoms. ETE in both childhood and adulthood relative to other trauma exposure groups was associated with more trauma symptoms, but only at low levels of awareness. There were no differences in trauma symptoms across trauma exposure groups at high levels of awareness. This suggests that low levels of acting with awareness may worsen trauma symptoms among women with a history of trauma. Subsequent ETE may trigger trauma symptoms characterized by intrusive images, memories, and emotions associated with the original trauma [63, 86]. To cope with these types of intrusions, individuals may attempt to suppress the unwanted thoughts or emotions. However, these attempts may further encourage the intrusive thoughts and emotions to surface [87]. Low levels of acting with awareness may worsen these intrusive thoughts and emotions. Still, whereas some studies have also found that acting with awareness is related to trauma symptomatology [54, 88], others have not [89]. Although further research is needed, our findings suggest that acting with awareness may be a modifiable target for interventions to increase intrapersonal resources for women in SUD treatment who display trauma symptoms and have a history of ETE, especially women with ETE in both childhood and adulthood.

Similarly, the indirect effect of ETE on craving via trauma symptoms was also moderated by acting with awareness. While medium effect sizes were found for the association between ETE and craving via trauma symptomatology, findings revealed small effects sizes for the moderated indirect effects of acting with awareness on craving. This suggests that women who report ETE during both childhood and adulthood compared to other trauma exposure groups report more trauma symptoms and subsequently more craving, but only at low and average levels of awareness. For women who reported high levels of awareness, there was no difference in craving across trauma exposure groups. Again, this suggests that low levels of acting with awareness may serve as risk factor for craving via trauma symptom. These results are in line with previous meta-analytic findings indicating that the acting with awareness dimension of mindfulness disposition is more robustly associated with substance use [90] and negative affect [52] compared to other facets of mindfulness disposition [90]. The moderating effect of acting with awareness is considered small, which is congruent with previous studies [90]; however, it may have clinical significance because it can be easily integrated into existing treatment protocols; it may be related to other proximal factors other than trauma symptoms that may reduce craving and can be a modifiable target for intervention for women in SUD treatment, particularly women of color [91, 92]. That is, previous studies found that mindfulness programs compared to relapse prevention programs lead to lower drug use for minorities but not White treatment attendees [92]; and an unpublished study, using the present data, suggests that mindfulness-based treatment approaches may be beneficial for racial minorities, particularly Hispanic women [81].

Lastly, our analyses and subsequent findings should be viewed with caution. Future research replicating these results in larger samples should further examine these constructs as beneficial targets for treatment of women with SUD and a history of trauma. Nevertheless, our findings present initial evidence for the possible beneficial integration of strategies and practices that cultivate the acting with awareness facet of mindfulness disposition in interventions for women with SUD and a history of ETE.

## Implications

While preliminary, findings from this investigation have several clinical implications. Results suggest that a lack of acting with awareness, one of the dimensions of trait mindfulness, may act as a risk factor for more trauma symptoms, which in turn may increase craving and potentially relapse in women with SUD and a history of trauma. Although mindfulness disposition is a naturally occurring trait for everyone, it can be enhanced through mindfulness training, such as that provided by mindfulness-based interventions [93]. Furthermore, childhood trauma has developmental consequences including emotional dysregulation [27], leading to health-compromising behaviors and cognitive problems [94]. Thus, behavioral interventions for SUDs should include, as part of their screening process, the timing of such exposures. This information may assist in developing a more individualized treatment plan for women in SUD treatment programs with distinct ETE. Finally, more research is needed to fully understand the mechanisms underlying the relationship between acting with awareness and craving. Thus, it is important for longitudinal studies to analyze the moderating effects of acting with awareness on substance craving.

## Limitations

Findings from this investigation should be interpreted in light of its limitations. First, the cross-sectional design precludes conclusions about causal inferences and mediating effects. However, the independent variable of interest in the present study is collected retrospectively based on past exposure to traumatic events, while the mediator, moderator, and outcomes are collected based on a past 30-day window. Therefore, the foundation for a temporal ordering is present and adds support for the current design [95, 96]. Nevertheless, we frame our findings in terms of indirect effects and suggest that the present study findings provide theoretical contributions and lay the foundation for further longitudinal mediation analysis of the key study constructs. Second, although we used validated self-report measures commonly used in the field, in-person interviews are subject to recall and social desirability bias. Third, our trauma exposure variable did not account for severity or number of traumatic events, which could also affect trauma symptoms and craving. Fourth, although the total mindfulness scale had good internal consistency, some mindfulness subscales did not display good internal consistency. Fifth, our sample size is considered small for a moderated indirect effects model analyses and could result in underreporting of significant findings. Still, our analysis detected some significant findings that provide possible future research on craving among women in SUD treatment. Sixth, the model may not include contextual factors that may elicit craving. Lastly, our findings may be limited to women in substance use treatment and lack generalizability to other populations.

## Conclusion

The present study findings contribute to our understanding of the associations among developmental timing of ETE, trauma symptoms, and craving and the role of trait mindfulness in these associations. Results show that ETE in both childhood and adulthood is associated with more craving via trauma symptoms and that low levels of acting with awareness may serve as a risk factor for more trauma symptoms and subsequently, more craving. Interventions that promote acting with awareness, such as mindfulness-based interventions, may be beneficial for women in SUD treatment in reducing craving.

## Data Availability

Data and materials are available with prior approval from the principal investigator (Dr. Hortensia Amaro) based on available time and resources.

## Abbreviations

ETE: Exposure to Traumatic Events
SUD: Substance Use Disorder
PTSD: Post Traumatic Stress Disorders
DSM: Diagnostic and Statistical Manual of Mental Disorders
HIPAA: Health Insurance Portability and Accountability Act
